# Multiplexed Enzyme Activity Microscopy by Semi-quantitative, Ratiometric Enzyme Activity MALDI Mass Spectrometry Imaging

**DOI:** 10.1021/acsmeasuresciau.6c00070

**Published:** 2026-08-12

**Authors:** Hamidreza Hojjat, Petra Henklein, Akvile Haeckel, Grit Nebrich, Sylwia Handzik, Pedro Augusto Dantas de Moraes, Jing Guo, Justus Ramtke, Yubei He, Lynn Jeanette Savic, Oliver Seitz, Oliver Klein, Eyk Schellenberger

**Affiliations:** † Institute of Radiology, 9373 Charité–Universitätsmedizin Berlin, Corporate Member of Freie Universität Berlin and Humboldt-Universität zu Berlin, Charitéplatz 1, Berlin 10117, Germany; ‡ Institute of Biochemistry, Charité–Universitätsmedizin Berlin, Corporate Member of Freie Universität Berlin and Humboldt-Universität zu Berlin, Charitéplatz 1, Berlin 10117, Germany; § 522475 Imaging Mass Spectrometry Unit; Berlin Institute of Health at Charité-Universitätsmedizin Berlin, Augustenburger Platz 1, Berlin 13353, Germany; ∥ Berlin Institute of Health at Charité-Universitätsmedizin Berlin, Anna-Louisa-Karsch-Str. 2, Berlin 10178, Germany; ⊥ Institute of Chemistry, Humboldt-Universität zu Berlin, Brook-Taylor-Str. 2, Berlin 12489, Germany

**Keywords:** cancer, enzymes, imaging mass spectrometry, mass spectrometry, peptides, spatial enzymology

## Abstract

Enzymes are often tightly regulated and exist in inactive forms. Therefore, rather than mere presence, it is the enzyme’s activity that catalyzes cellular and tissue functions. However, multiplex activity mapping of enzymes in situ is particularly challenging. To address this, we introduce multiplexed enzyme activity MALDI–MSI (MEA–MSI), a ratiometric, semi-quantitative method that allows multiplex in situ visualization of enzyme activities in zinc-fixed tissue sections. To accomplish this, a library of 31 MEA–MSI substrates targeting proteases, kinases, histone acetyltransferases, and glycosyltransferases was synthesized. Each substrate is designed so that the masses of these substrates and their enzymatic products are distinct, enabling their simultaneous pixel-wise detection within tissue sections. A ratiometric calculation procedure was used to estimate the relative enzyme activity and compensate for tissue-dependent ionization bias, which is a general problem in MALDI–MSI. MEA–MSI was applied to healthy mouse liver, a murine hepatocellular carcinoma (HCC) model, and an incomplete microwave-ablated (iMWA) rabbit VX2 liver tumor model. The resulting activity maps revealed distinct regional enzyme activity patterns across tumor and normal tissues. Correlation and network analyses showed related enzyme activities and related groups, while unsupervised clustering and Uniform Manifold Approximation and Projection (UMAP) analysis reproduced histological patterns. Because MEA–MSI relies on enzymatic amplification, it is not restricted to the detection of highly abundant enzymes and enables multiplexed, spatially resolved readouts of enzyme activity in situ, providing a framework for comparative spatial enzymology.

## Graphical abstract

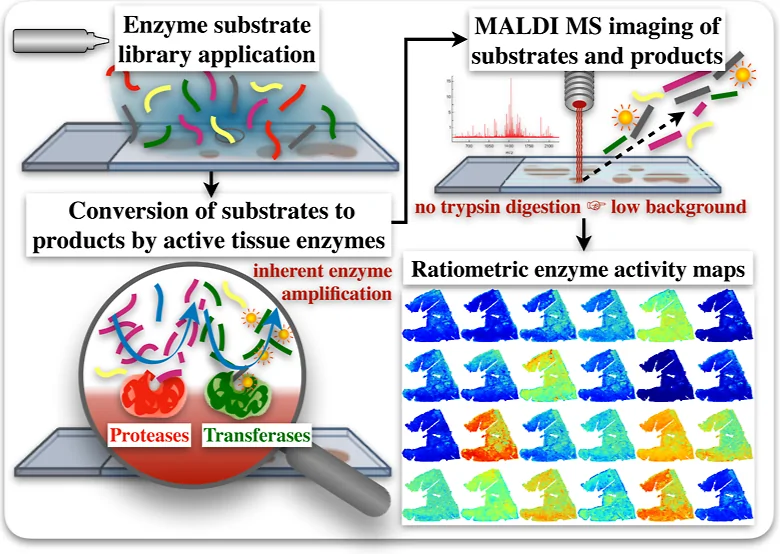

## Main

Enzymes are biological catalysts essential for metabolism, signaling, and regulation, which define the functional and physiological states of tissues. Dysregulated enzyme activity contributes to a variety of pathologies, including cancer, liver disorders, and metabolic diseases. Their distribution within tissues is often spatially heterogeneous, such as in the zonated architecture of the liver, where local variations in enzyme abundance and activity drive regional metabolic differences and influence disease progression. Despite their importance, such spatial patterns still remain difficult to resolve comprehensively.^1–3^

Imaging techniques such as immunohistochemistry (IHC), imaging mass cytometry (IMC), and matrix-assisted laser desorption/ionization mass spectrometry imaging (MALDI–MSI) have provided spatial mapping of proteins and metabolites within tissue sections.^4–8^ However, these abundance-based approaches report molecular presence rather than catalytic functions of enzymes. Protein levels frequently fail to reflect enzymatic activity due to post-translational regulation and inhibition.^9,10^ This is particularly relevant in the context of spatial proteomics and spatial transcriptomics, which map molecular abundance but do not directly capture catalytic activity. Moreover, ionization efficiency in MALDI–MSI influences intensity maps, making quantification challenging.^11,12^ Consequently, nonactivity-based approaches cannot fully capture the functional proteome that defines tissue physiology.

Activity-based enzyme imaging techniques address these limitations by directly measuring catalytic conversion rather than protein abundance. Methods such as in situ zymography and fluorogenic or quenched probes visualize enzyme activity with high sensitivity but are limited by autofluorescence, spectral overlap, and limited multiplexing.^13–15^ Microscopy-based activity-based protein profiling (ABPP) offers improved spatial resolution but remains low-plex and semiquantitative.^16–19^ Using zinc-based fixation protocols, which preserve both morphology and enzymatic function, activity assays on fixed sections are possible.^20–22^ Meanwhile, MALDI–MSI-based enzyme assays provide a label-free approach to detect substrate-product pairs directly on tissue; however, previous studies have been restricted to one or two natural substrates and mainly specific metabolite conversions.^23–25^ Until recently, there was no existing approach that could achieve multiplexed, spatially resolved mapping of enzyme activities within a single tissue section.

Recently, we introduced a multiplex enzyme activity imaging approach using synthetic peptide substrate libraries for MALDI–MSI on zinc-fixed tissues.^26^ In this proof-of-concept study, each peptide was designed to generate distinct mass signatures upon enzymatic modification, enabling the multiplexed detection of enzyme activity within tissue sections. This study demonstrated the feasibility of activity-based imaging using MALDI–MSI but was mainly limited to proteases and healthy kidney tissue. These promising results highlighted the potential of synthetic peptide substrate library application to expand spatial enzymology beyond conventional single-enzyme assays and MALDI–MSI.

Extending our previous study, we developed multiplexed enzyme activity by MALDI–MSI (MEA–MALDI–MSI or MEA–MSI), a platform that simultaneously measures the activity of multiple enzyme classes directly on tissue sections (Figure 1). We used a 31 synthetic peptide substrate library targeting proteases, kinases, histone acetyltransferases, and glycosyltransferases. Each substrate is designed for distinct mass signatures upon conversion by endogenous enzymes, enabling multiplexed detection. To compensate for regional ionization bias and generate semi-quantitative enzyme activity maps, ratiometric quantification by fractional conversion of product to substrate maps is performed.^27,28^ MEA–MSI benefits from inherent enzymatic amplification; during the incubation period, a single catalytically active enzyme molecule can convert multiple substrate molecules into detectable product molecules. Similar to the amplification principle used in enzyme-linked immunosorbent assays (ELISA), this activity-based signal generation can increase sensitivity for low-abundance but active enzymes that might remain difficult to detect by conventional abundance-based MALDI–MSI or abundance-based spatial omics approaches. Using zinc-fixed tissues and MALDI–MSI, we mapped enzyme activities in healthy mouse liver, a murine hepatocellular carcinoma (HCC) model, and an incomplete microwave-ablated (iMWA) rabbit VX2 liver tumor model.

**Figure 1:**
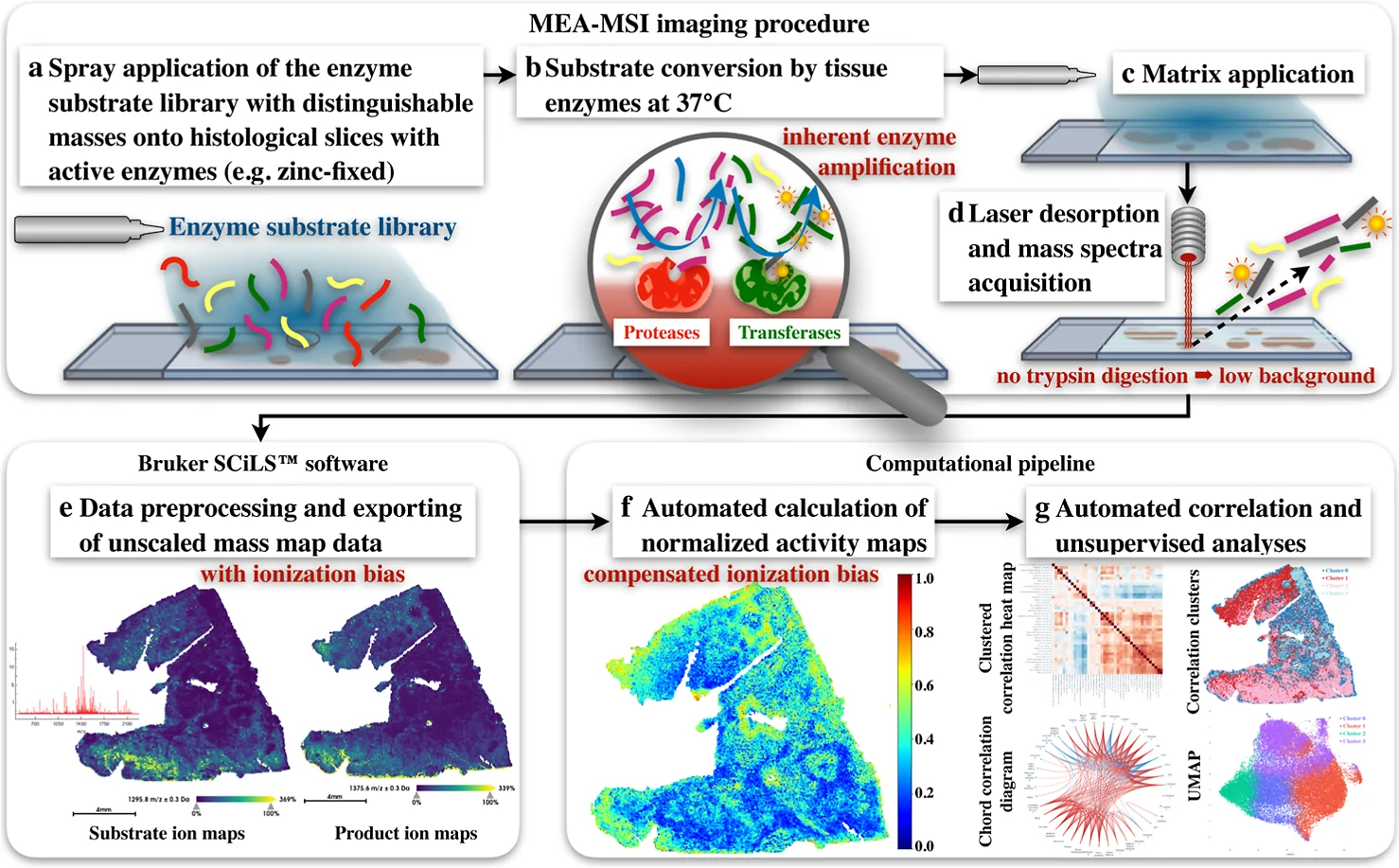
Workflow for MEA–MALDI–MSI to map multiplexed enzymatic activities in tissue. (a) Thirty-one high-purity natural protein-derived peptide substrates were synthesized and mixed to target four enzyme classes: proteases, kinases, histone acetyltransferases (HATs), and glycosyltransferases (GTs). Zinc-fixed, paraffin-embedded tissue sections were obtained from rabbit/mouse cancerous and normal mouse liver tissues. The peptide library is uniformly sprayed onto the tissue sections. (b) Endogenous enzymes convert substrates to products. This results in time-dependent, enzyme-amplified product accumulation, even for low-abundance enzyme activities. (c) The matrix (α-cyano-4-hydroxycinnamic acid, CHCA) was spray-applied after incubation. (d) MALDI imaging was performed (50 μm raster on a RapifleX MALDI–TOF instrument). (e) MALDI–MSI data sets were imported into SCiLS Lab, baseline-corrected, and preprocessed (example: raw ion-intensity maps of the cha 154 substrate (*m*/*z* 1295.8) and its phosphorylated product cha 154.P1 (*m*/*z* 1375.6) acquired after 30 min of incubation) and exported as CSV. (f) The Python scripts generate normalized spatial activity images (pixel-wise ratiometric activity maps calculated as *I*_product_/(*I*_product_ + *I*_substrate_) according to eq 1). The values ranged from 0 (no enzymatic conversion) to 1 (complete enzymatic conversion). (g) Based on the normalized activity maps, Python scripts generated heatmaps, UMAP embeddings, and correlation/network visualizations.

MEA–MSI bridges the gap between molecular abundance and enzymatic function by enabling spatially resolved semi-quantitative mapping of diverse enzymatic activities in situ in tissues. We define our measurements as “semi-quantitative”, denoting the relative substrate conversion across pixels and time points under fixed assay conditions; absolute kinetic parameters, such as kcat or km, are currently not inferred.

## Results

### Substrate Library and MEA–MSI

Thirty-one naturally derived peptide substrates were synthesized with a median reverse-phase high-pressure liquid chromatography (RP-HPLC) purity of 99.3% (range 95.6–99.9%) and combined to prepare a multiplex enzyme activity substrate library mixture used for MEA–MSI (Table 1, Supplementary Table S1). MEA–MSI was performed on healthy mouse liver, a murine hepatocellular carcinoma (HCC) model, and incomplete microwave-ablated (iMWA) rabbit VX2 liver tumor. Tissue sections were mounted on ITO-coated slides, homogeneously spray-applied with the peptide substrate mixture, and incubated for 30 min (or 60 min, see supplements) at 37 °C in a humidity chamber for the enzyme conversion of substrates to products. After matrix application, MSI of the tissue slides was performed with a resolution of 50 μm × 50 μm.

**Table 1:** | Thirty-One-Peptide MEA Library^a^

peptide substrate ID	target enzyme	enzyme class	substrate sequence and modification site	substrate source (UniProt)
**cha 151_1**	Calpain-1	protease	GQGPGPK ***|RGT*** EPKV	Q15113
**cha 152_1**	Thimet oligopeptidase		PGFSP ***FRSS|RIGE*** I	P01042
	Neurolysin		PGFS ***PFRS|SRIG*** EI	
	Plasma Kallikrein		PGF ***SPFR|SSRI*** GEI	
	Endothelin-converting enzyme 1		P ***GFSP|FRSS*** RIGEI	
	Kallikrein-related peptidase 5		PGFSPF ***RSSR|IGEI***	
**cha 153_1**	MMP2		VSR ***LRAY|LLPA*** PPA	P17936
	Cathepsin D		VS ***RLRA|*** Y ***LLP*** APPA	
	Thrombin		V ***SRLR|AYLL*** PAPPA	
	Plasmin		***VSR|LRAY*** LLPAPPA	
**cha 154_1**	Matriptase-2		***NKRR|G*** SVPILR	P16452
	PKA	Kinase	NKR RGS*VP ILR	
**cha 155_1**	Paracaspase	Protease	SFEADE ***PDSR|A*** GVKWKN	O54786
	Cathepsin S		SFEADEPDSR ***AGVK|*** W ***K*** N	
**cha 156_1**	Cathepsin B		***RILL|*** DPGALPALQNPPIRGG	Q03167
**cha 157_1**	Kallikrein-related peptidase 12		KTKEGVLYVG ***SKTK|*** EG	P37840
**cha 158_1**	MMP3		NAPKR ***PPSA|FFLF*** CSEH	P26583
	MMP2		NAPKR ***PPS|A*** FFLFCSEH	
	Cathepsin B		***N|APK*** RPPSAFFLFCSEH	
**cha 159_1**	MMP9		RVET ***LSQV|EVIL*** QQSAA	P02469
	Cathepsin L		RVETLSQVEVILQ ***QSA|A***	
**cha 160_1**	DESC1 peptidase		GA ***KFR|*** I ***S*** LGLPVG	P62829
	MMP3		***GAK|FRIS*** LGLPVG	
**cha 161_1**	ERK-1	Kinase	E PLT*PSG EAPPFR	P00533
**cha 162_1**	Ribosomal protein S6 kinase beta-1	Kinase	VRR FNT*AND DPFR	P04040
	ADAMTS5 peptidase	Protease	***VRR|*** FNTANDDPFR	
**cha 163_1**	PKC	Kinase	KRK QIS*VR GLPFR	P11217
	Cathepsin L	Protease	KRKQI ***SVR|G*** LPFR	
	Cathepsin B		***KRK|*** Q ***I*** SVRGLPFR	
**cha 164_1**	CDC2	Kinase	PST NSS*PVL KPFR	Q14674
	Cathepsin B	Protease	***PST|*** NS ***S*** PVLKPFR	
**cha 166_1**	Aurora kinase A	Kinase	RPQ RAT*SNV FPFR	P24844
	Matriptase-2	Protease	***RPQR|*** A ***T*** SNVFPFR	
	Meprin beta subunit		***RPQ|*** RATSNVFPFR	
**cha 167_1**	Parl peptidase		***KRS|A*** EGQVDGADDLSTSD	Q03164
**cha 168_1**	Caspase-3		LFT ***DFPD|KAVK*** FLNKE	P09394
**cha 169_1**	Granzyme M		YGA ***AAGL|V*** PGGPGFGPGV	P15502
	MMP9		YG ***AAAG|L*** VPGGPGFGPGV	
	Elastase-2		YGAAAGLVPGG ***PGFG|*** PGV	
**cha 170_1**	Cathepsin K		***LGTN|*** PLKSSGIENGA	P07585
**cha 171_1**	Granzyme A Granzyme K		KRVHMTW ***VQPR|*** VLD	Q9UNM6
**cha 172_1**	Cathepsin F		V ***YASQ|*** ETFGMKLSV	Q9GZQ8
**cha 173_1**	Cathepsin E		RVSVQLEA ***SPAF|LAVP*** VEK	P01023
**cha 174_1**	HistoneAcT	Histone acetyltransferase	SG ***RGK°GGK*** GLGKGGAKR	P62805
	MMP3	Protease	SGRGKGGK*G* **LGK|**G ***G*** AKR	
	Granzyme A		SGRGK ***GGK|G*** LGKGGAKR	
	Granzyme A		SG ***RGK|G*** GKGLGKGGAKR	
	MMP2		***SGR|G*** KGGKGLGKGGAKR	
**cha 175_1**	HistoneAcT	Histone acetyltransferase	***RTK°QTA*** RKSTGGKAPR	Q6NXT2
	Cathepsin B	Protease	RT ***KQTA|*** RKSTGGKAPR	
	Cathepsin L		RTKQT ***ARK|S*** TGGKAPR	
	Matriptase-3		RTK ***QTAR|KSTG*** GKAPR	
**cha 176_1**	HistoneAcT	Histone acetyltransferase	PD ***PAK°SAP*** APKKGSKKAV	P23527
	Granzyme A	Protease	PDPAKSA ***PAPK|KGSK*** KAV	
	Cathepsin L		P ***DPAK|SAPA*** PKKGSKKAV	
**cha 184_1**	Caspase-1		DDLF ***FEAD|GPKQ*** MKCSFQ	P01584
	Cathepsin L		DDLFFEADGP ***KQMK|*** CSFQ	
**cha 189_1**	GalNAc-T1	Glycosyl transferase	***GTT″PSPVP*** TTSTTSAPT	P98088
	MMP3	Protease	GTTPSPVPTTST ***TSAP|*** T	
	MMP2		GTTPS|PVPTTSTTSAPT	
**cha 190_1**	GalNAc-T1	Glycosyl Transferase	GAG ***FFPT″PGPA*** GAGK	
	MMP9	Protease	GAGFFPTP ***GPAG|A*** GK	
	Cathepsin S		GAGFFP ***|TPGP*** AGAGK	
	MMP3MMP7MMP12		GAGFFPTPGP ***AGAG|*** K	
**cha 192_1**	Caspase-3		RKG ***DEVD|GVDE*** VAK	P09874
	Caspase-7		R ***KGD|*** EVDGVDEVAK	
	Cathepsin E		RKGDEVD ***GVDE|*** VAK	
**cha 193_1**	SIK1	Kinase	NKR RGS*VP ILPFR	P16452
	Matriptase-2	Protease	***NKRR|G*** SVPILPFR	
**cha 194_1**	AKT1	Kinase	RLR RLST*KY RPFR	Q05209
	Site-1 peptidase	Protease	RL ***RRLS|*** TKYRPFR	
	Furin		***RLRR|*** LSTKYRPFR	

a Columns list the peptide substrate ID, target enzymes, enzyme class, peptide substrate (recognition) sequence with cleavage site (“|”) and post-translational modifications (*′ = phosphorylation; “°” = ε-acetyl-Lys; “″” = O-GalNAc), and UniProt accession of the parent protein.

A substrate or product ion was recorded as detected when its theoretical *m*/*z* (±0.3 Da) aligned with the orthogonal-matching-pursuit feature (OMP) list produced by SCiLS Lab software with default setting,^29^ which is designed to find those peaks inside a spectrum encoding relevant information (detected in >1% of spectra). After 30 min of enzyme conversion, 30/31 substrates (96.8%) (Table 1) were detected; the only undetected substrate was cha 157_1. Substrate/product-ion pair detection at this time point was grouped as follows: class A (substrate and product detected), 58; class B (substrate detected, product not detected), 24; class C (product detected, substrate not detected), 1; and class D (neither substrate nor product(s) was detected),0 (Supplementary Table S2). The total number of detected product ions exceeded the number of substrates because several protease substrates generate two cleavage products, and some substrates are designed to be recognized by different enzymes, resulting in distinct product ions. After 60 min of incubation, the substrate coverage was slightly reduced to 28/31 (90.3%), with cha 157_1, cha 176_1, and cha 184_1 not detected, and the product-ion distribution shifted to *A* = 58, *B* = 19, *C* = 1, and *D* = 5. Detection was also evaluated separately for each tissue model and showed the same overall substrate/product detection pattern as the combined tissue section analysis.

To assess discriminative performance, receiver operating characteristic (ROC) analysis was performed in SCiLS Lab, comparing spectra from substrate-treated sections to spectra from control sections without substrate application.^30–32^ The individual ROC plots against the unsprayed control, ion-intensity maps, spectral data, detection details, and AUC values for each ion are provided in Supplementary Figures S1–S4 and Supplementary Table S2. Overall, MEA–MSI achieved near-complete substrate detection with high discriminative performance at 30 min, whereas longer incubation increased product coverage at the expense of lower substrate detectability.

### Ratiometric Activity Normalization

Pixel-to-pixel differences in MALDI ionization efficiency limit direct comparisons of enzyme activity in raw MALDI–MSI images. For example, raw images of the PKA substrate cha 154 (*m*/*z* 1295.8) and its phosphorylated product cha 154.P1 (*m*/*z* 1375.6) displayed partly overlapping ion intensity distributions instead of the expected complementary patterns (Figure 1e). A largely similar ionization pattern was observed across all substrate and product ion maps (Supplementary Figures S1,S2), indicating similar modulation of the spatial ionization efficiency. However, product-ion distributions are also concentration-dependent and reflect local substrate conversion; therefore, product maps cannot be attributed to ionization effects alone. To address this, pixel-wise ratiometric normalization of substrate and product pairs was applied, which was defined as the product intensity divided by the sum of the product and substrate intensities, yielding values between 0 and 1 (eq 1).^27,33^ This approach is supported by previous mass spectrometry-based enzyme assays using substrate to product conversion readouts. However, because substrate and product ions can differ in intrinsic ionization efficiency due to molecular weight, sequence, structure, or modification state, the resulting ratio is interpreted as a semiquantitative activity readout rather than an absolute measure of enzymatic conversion. As a result, the ratio maps display distinct activity patterns across all three analyzed tissue probes (Figure 1f, iMWA rabbit VX2 liver tumor tissue section shown) that were hardly visible in the raw intensity maps. All subsequent activity maps, as well as the correlation and clustering analyses, used these normalized images.

1 $$Ratio=\frac{I_{product}}{I_{product}+I_{substrate}}$$

For substrates yielding multiple products, ratios were calculated separately for each parent substrate/product pair. Therefore, each ratio represents the apparent conversion into the selected product ion rather than total conversion of the parent substrate into all possible products.

### Enzyme Activity Maps

Ratiometric activity maps were generated from raw ion maps acquired after 30 min of incubation at 37 °C, of which 36 representative activity maps are shown in Figures 2 and 3. One representative product with a high signal intensity per enzyme was included in the panel and further analyzed. For protease substrates, which can generate two cleavage products, only one product was retained to avoid overweighing these reactions in downstream analyses. In addition, some substrates can be converted by multiple enzymes, yielding distinct products. Consequently, a total of 36 representative enzyme activity maps were generated from the 31-substrate library. Complete activity maps for 30 and 60 min time points (jet color scale and raw) are shown in Supplementary Figures S5–S12.

**Figure 2:**
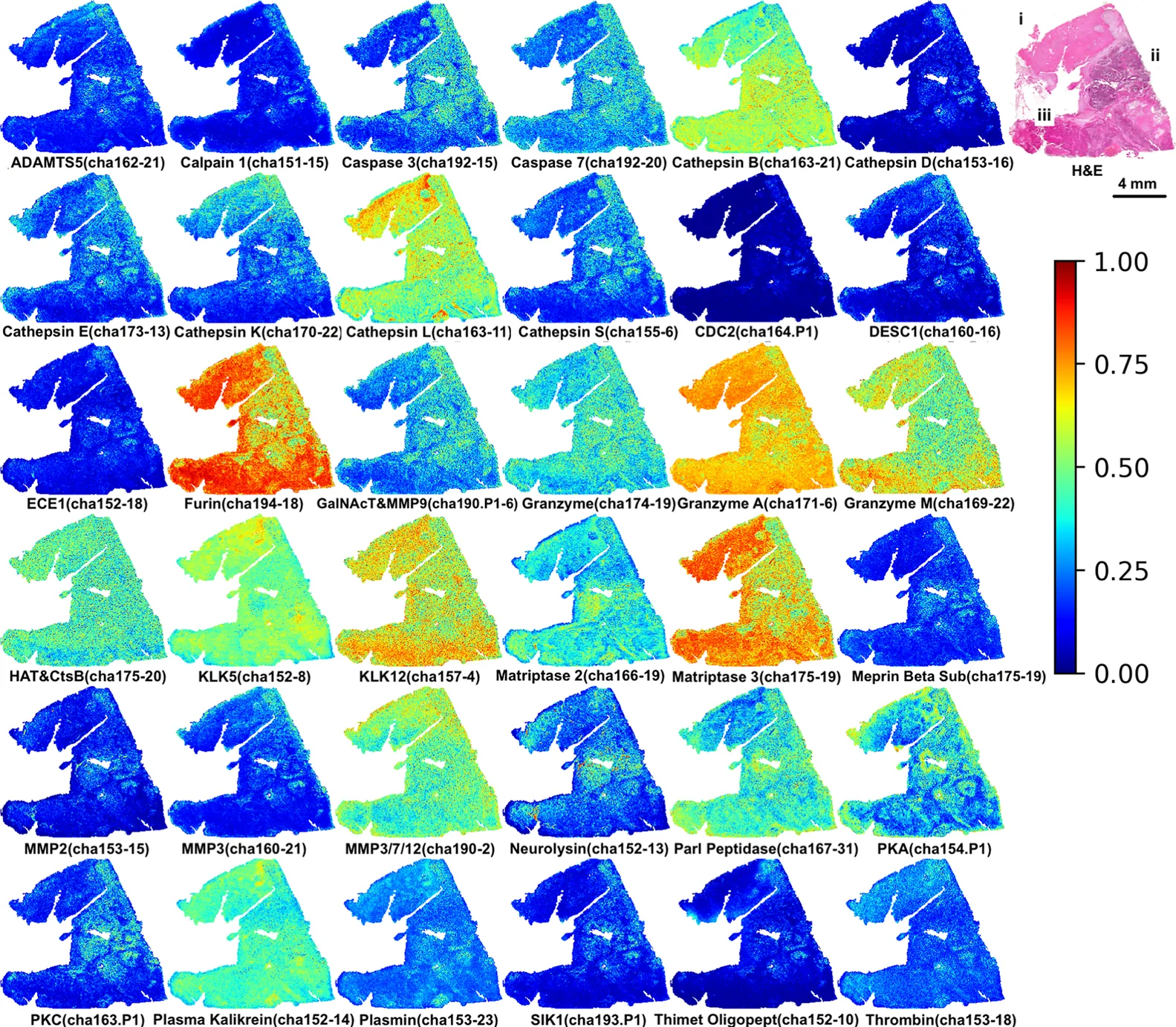
Ratiometric enzyme activity maps after 30 min of incubation in the iMWA rabbit VX2 liver tumor model. The iMWA rabbit VX2 liver tumor atlas comprises one hematoxylin and eosin (H&E) overview image (bottom right corner, H&E section with tumor-adjacent liver (upper zone), a mixed viable tumor/necrotic region (middle zone), and mixed iMWA-altered tissue (lower zone)) together with 36 normalized activity maps scaled from 0 to 1 displayed using a jet colormap. Scale bar: 4 mm (shown in H&E). Maps are labeled with the target enzyme and peptide product identifier (see Table 1; colored and raw map stacks provided as supplements).

**Figure 3:**
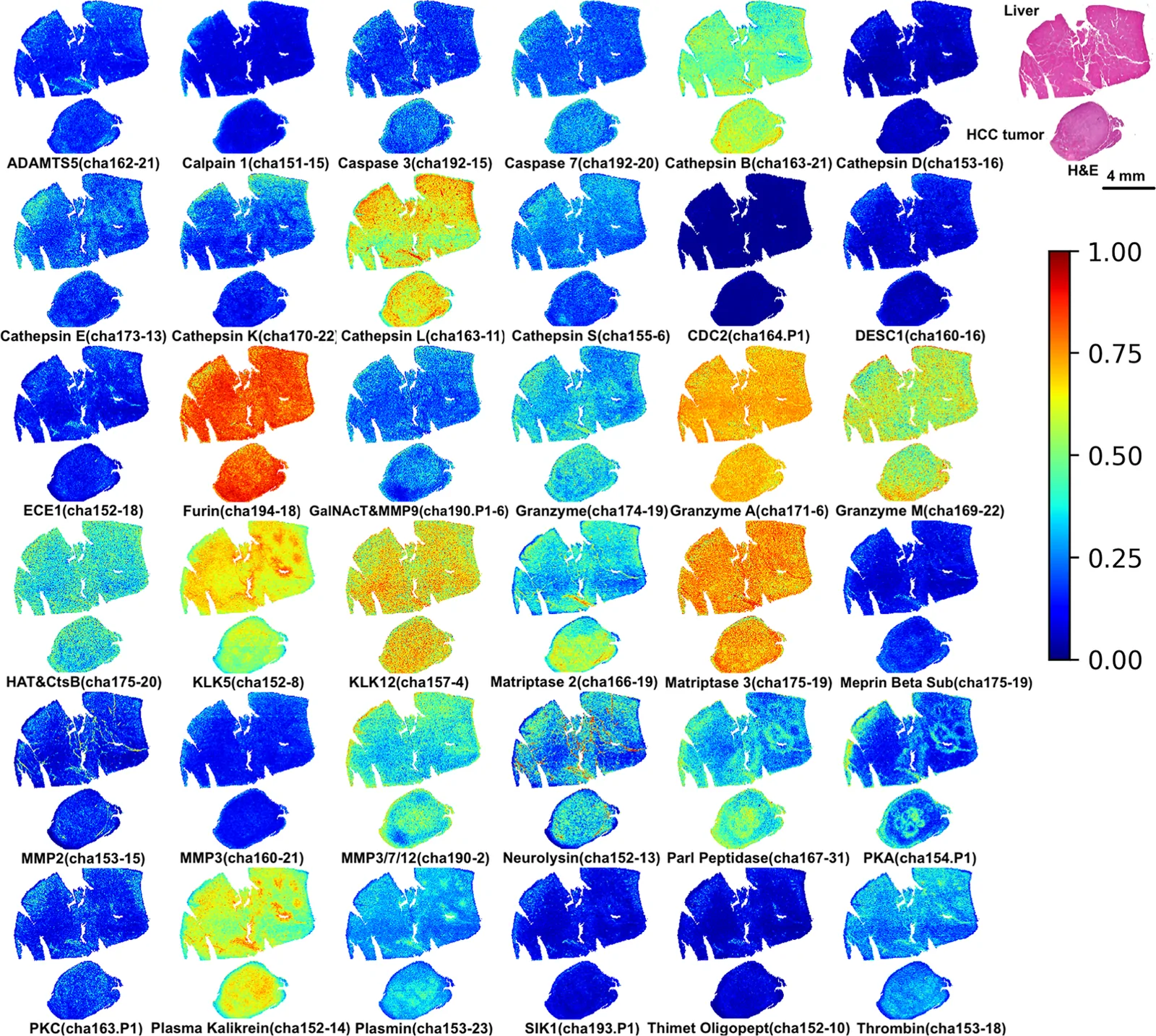
Ratiometric enzyme activity maps after 30 min of incubation in mouse liver models. Mouse liver atlas comprises one H&E overview image (down right corner, normal liver (up) and hepatocellular carcinoma (down) sections) followed by 36 normalized activity maps scaled from 0 to 1 displayed using jet colormap. Scale bar: 4 mm (shown on H&E). Maps are labeled with the target enzyme and peptide product identifier (see Table 1, colored and raw map stacks provided as supplements).

In the iMWA rabbit VX2 liver tumor section (hematoxylin and eosin stain, H&E, Figure 2 and Supplementary Figure S13,14), three histological zones were visible: tumor-adjacent liver (upper), a region of both viable tumor and necrotic tissue (middle), and mixed iMWA-altered tissue (lower). The 36 activity maps showed different visual patterns (Figure 2). Cathepsin L activity was predominantly observed in the tumor-adjacent liver. Sixteen enzymes; calpain-1, caspase-3, caspase-7, cathepsin D, cathepsin E, cathepsin S, DESC1 peptidase, CDC2, PKC, GalNAcT, SIK1, Meprin-β, endothelin-converting enzyme-1, thimet oligopeptidase, MMP-2, and MMP-3, were found predominantly in the tumor/necrotic zone. No enzyme activity was observed exclusively in the mixed iMWA-altered tissue. Cathepsin K and ADAMTS5 peptidase showed extended patterns from the tumor-adjacent liver into the tumor/necrotic zone. The distribution of Parl peptidase spanned both the tumor/necrotic region and the mixed iMWA-altered tissue. Furin, Granzyme A/K, Granzyme M, KLK5, KLK12, matriptase-3, Plasma Kallikrein, and Thrombin appeared in tumor-adjacent liver and mixed iMWA-altered tissue but were lower in the middle zone. Finally, cathepsin B, granzyme A, matriptase-2, neurolysin, HAT, plasmin, and PKA had separate distribution patterns that did not fit into the previous groups.

The mouse liver activity maps included sections of normal tissue (Figure 3, upper tissue, and Supplementary Figures S13,14) and HCC (Figure 3, lower tissue, and Supplementary Figures S13,14). Executioner caspases (caspase-3 and caspase-7) and the proteases matriptase-2 and Parl peptidase showed visually higher ratiometric activity signals in the HCC section than in the normal liver section, although the differences were modest. Cathepsin K, cathepsin S, and KLK5 were more active in normal liver. The other enzymes, such as furin, cathepsin L, several MMPs, and granzymes, showed similar activity in both tissues. These activity maps, which were interpreted descriptively, could highlight the distinct spatial enzyme activity in rabbit and mouse liver tissues, providing a foundation for comparative and integrative analyses.

To investigate whether the abundance of the cosubstrate ATP could limit the enzyme conversion of the kinase substrates, we performed an MEA–MSI experiment on a mouse HCC section with or without ATP addition for 30 min under identical conditions. PKA (cha 154.P1) and CDC2 (cha 164.P1) product-ion peaks were detected with or without additional ATP supplementation. Ratiometric activity maps acquired from these sections showed a consistently higher enzymatic signal across the tissue in the ATP-supplemented condition, while showing a similar spatial distribution (Figure 4b,d). The pixel-wise ratiometric activity distributions shifted toward higher values in the presence of ATP, with statistically distinct medians (*p* < 0.0001 by Mann–Whitney; Figure 4c,e). This observation was pronounced in regions with high kinase activity, where ATP depletion was more likely to have a limiting effect. This ATP-dependence supports the notion that the observed activity originates from ATP-dependent enzymatic phosphorylation, validating the biological specificity of the ratiometric signal.

**Figure 4:**
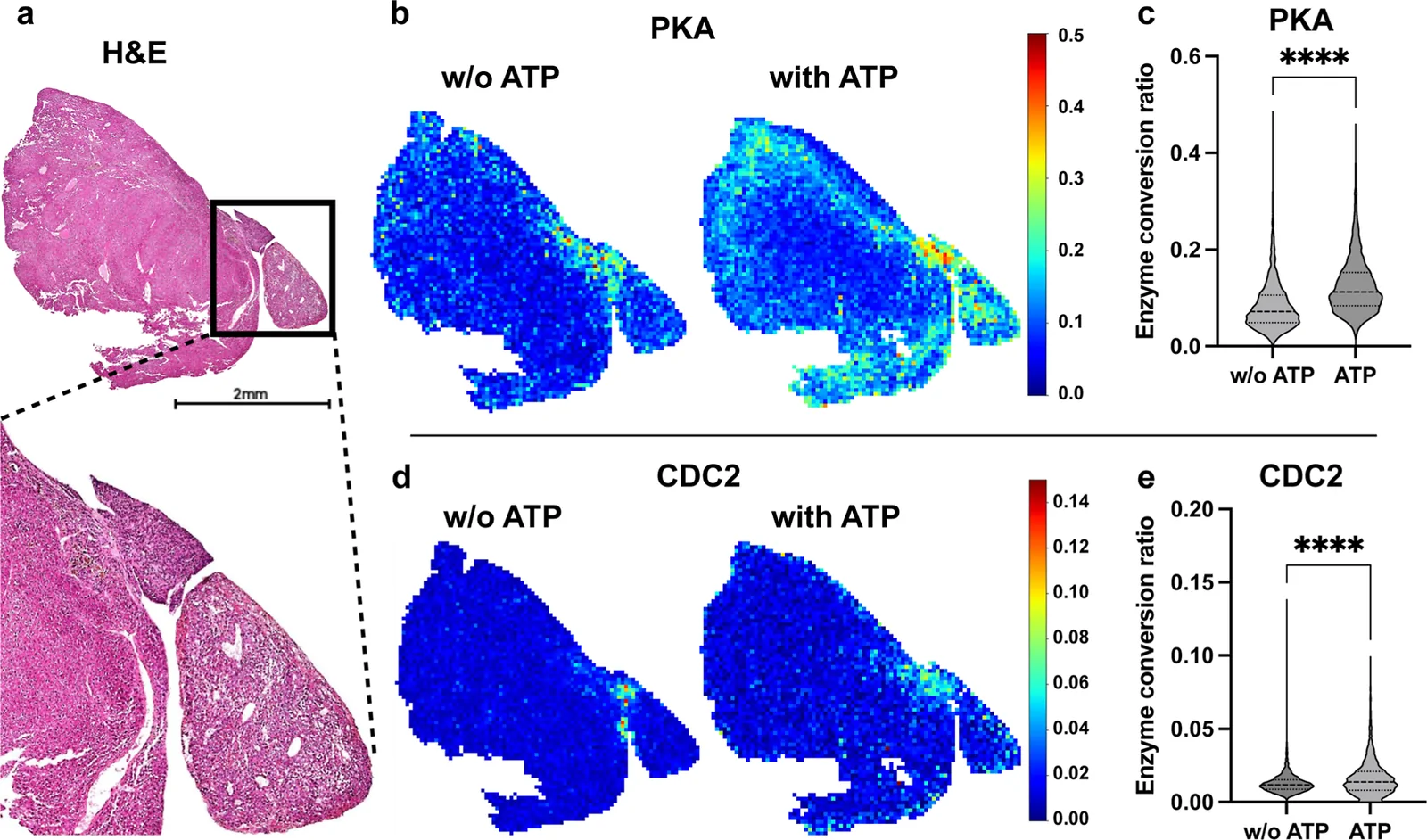
ATP-dependence of kinase activity maps MEA–MSI maps were generated from mouse liver tissue with adjacent HCC tumor tissue (box) with and without the addition of ATP to the applied substrate library mix for 30 min of incubation. (a) H&E staining of mouse HCC section. (b) Ratiometric activity map of PKA. (c) Pixel-wise ratiometric activity distributions shown as violin plots (Mann–Whitney *p* < 0.0001) for PKA. (d) Ratiometric activity map of CDC2. (e) Pixel-wise ratiometric activity distributions shown as violin plots (Mann–Whitney *p* < 0.0001) for CDC2.

### Reproducibility of MEA–MSI

To assess the reproducibility of MEA–MSI, we compared substrate and product ion detection across technical replicate preparations and across independent animals for each tissue model. From the original animal (specimen 1), two additional replicate sections (specimens 2 and 3) were prepared using a different sprayer and matrix supplier than the original measurement (H&E, Supporting Information, Figures S15, S16). A further section from a second animal (specimen 4) was prepared under conditions matched to the original measurement (H&E, Supporting Information, Figure S17). Detection of each substrate and product ion was scored across preparations and animals (Supporting Information, Tables S3 and S4).

Across the original preparation and the two adjacent replicate preparations from the same animal (same tissue block), 21 of 31 substrates and 41 of 83 product ions were detected in all three preparations. Several ions were detected only in the original preparation, including 8 substrates and 20 products, whereas 5 products were detected only in the replicate preparations. Discordance between the two replicate preparations was limited to one substrate and two product ions, indicating that ion detection was largely consistent when the preparation conditions were held constant. These findings suggest that part of the variability in substrate and product detection was associated with the change in preparation batch, including sprayer and matrix (Figure 5a,b).

**Figure 5:**
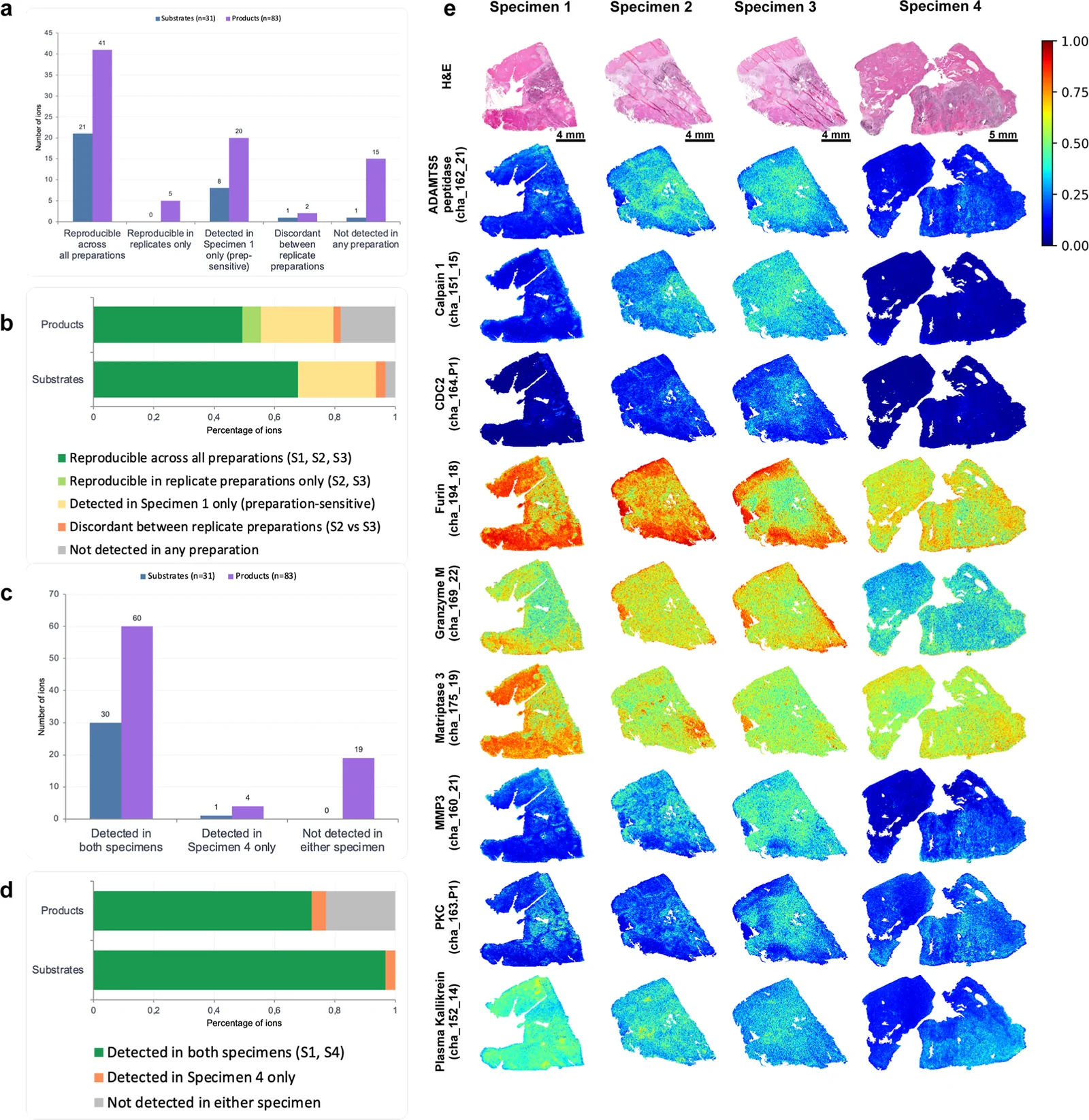
Reproducibility of MEA–MSI across replicate preparations and independent animals. (a and b) Detection reproducibility across the original animal and its two adjacent replicate preparations (specimens 1–3), prepared using a different spraying device and matrix supplier: number (a) and proportion (b) of substrate (*n* = 31) and product (*n* = 83) ions in each reproducibility category. (c and d) Detection similarity across two independent animals processed under matched preparation conditions (specimen 1 vs specimen 4): number (c) and proportion (d) of substrate and product ions in each detection category. (e) Representative ratiometric activity maps for nine enzymes shown across the four specimens, with the corresponding H&E-stained sections shown in the top row. Rows indicate enzymes and columns indicate specimens.

Across two independent animals processed under matched preparation conditions, the detection was highly similar for substrates and product ions. In total, 30 of 31 substrates and 60 of 83 product ions were detected in both animals, whereas only one substrate and four product ions were detected in a single animal. The remaining product ions were not detected in either of the matched preparations (Figure 5c,d).

The corresponding ratiometric activity maps showed similar spatial distributions between the two independent animals processed under matched conditions (specimen 1 vs specimen 4) and between the two adjacent replicate preparations generated under the same preparation conditions (specimen 2 vs specimen 3). In contrast, comparisons between the original section and its adjacent preparations (specimen 1 vs specimens 2–3) showed both conserved and divergent spatial patterns, depending on the enzyme. This is consistent with the preparation sensitivity observed in the ion-detection analysis (Table S3). Figure 5e shows examples of enzymes with conserved cross-animal and cross-batch activity patterns, whereas the complete set of ratiometric activity-map comparisons is provided in Supplementary Figures S18–S23.

### Enzyme Correlation Matrices and Networks

For each tissue probe, we computed pairwise Pearson correlations between the 36 ratiometric activity maps recorded at the 30 min incubation, which generated a 36 × 36 matrix with 630 unique activity correlation values that were visualized as heatmaps (Figure 6a,b). The corresponding correlation matrices for the 60 min incubation time point, along with interactive HTML heat maps for the complete set of enzymes, are provided in the Supporting Information.

**Figure 6:**
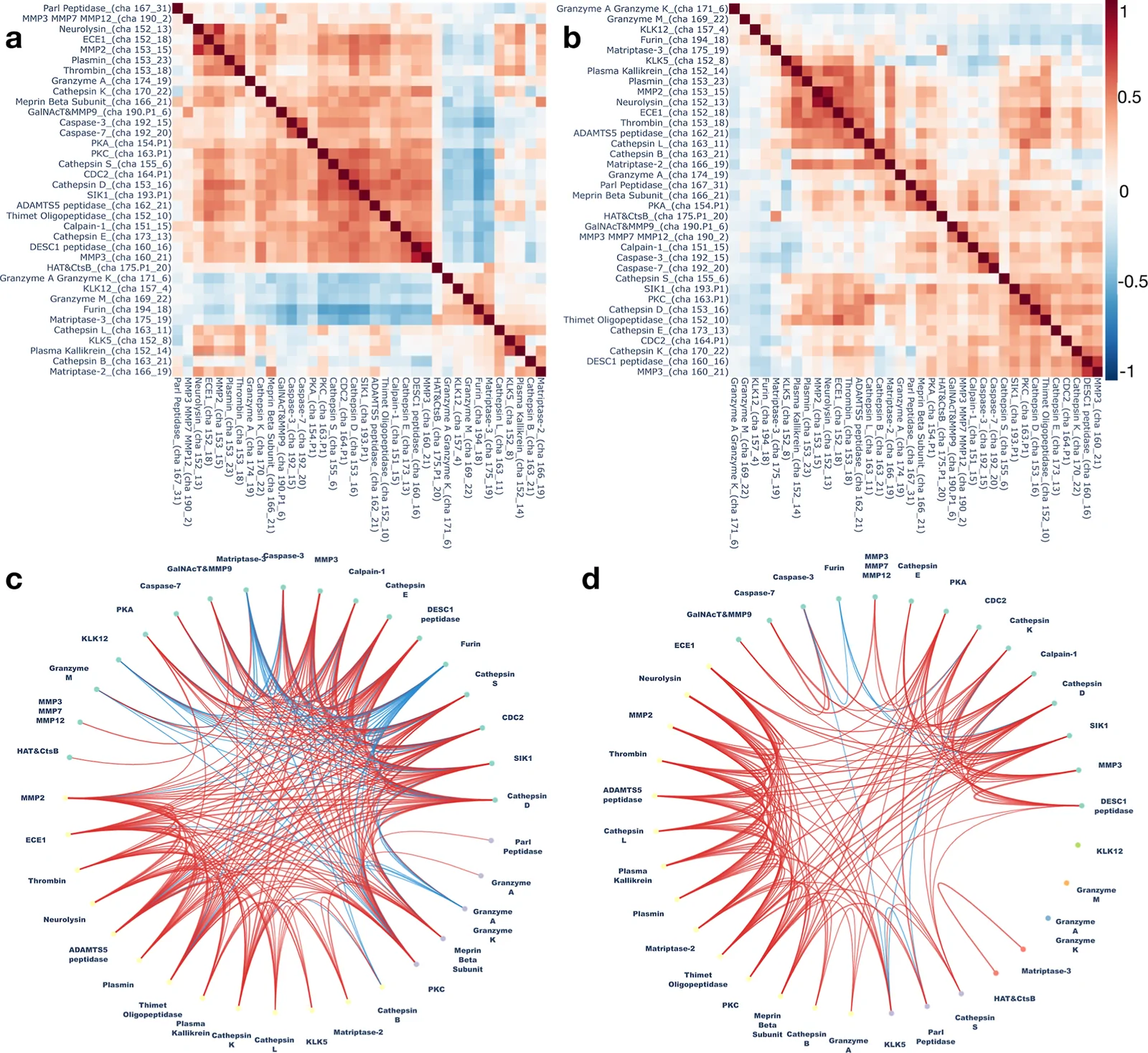
Correlation structure, chord network, and community structure of ratiometric enzyme activities (30 min incubation). (a) iMWA rabbit VX2 liver tumor: 36 × 36 Pearson correlation matrix of the ratiometric activity maps, ordered by average-linkage hierarchical clustering. The color scale spans −1 (blue) to +1 (red). (b) Mouse liver (normal and HCC): equivalent 36 × 36 correlation matrix. (c) iMWA rabbit VX2 liver tumor: chord diagram of the 36-node network constructed by retaining edges, where |*r*| > 0.30 from Pearson’s correlation matrix. Nodes are grouped (and colored) by communities detected with greedy modularity; link thickness is proportional to |*r*|. (d) Mouse liver (normal and HCC tissue). Network built using the same threshold. Interactive HTML versions are provided as a supplement.

In the iMWA rabbit VX2 liver tumor tissue, 200 enzyme pairs were positively correlated with *r* > 0.30, 53 were negatively correlated with *r* < – 0.30, and the remaining 377 were not substantially correlated with r between −0.30 and 0.30 (Figure 6a). For pairs with *r* > 0.30, the distribution had a median r of 0.435 (IQR 0.134); for pairs with r < – 0.30, the median was −0.365 (IQR 0.111); and for the remaining pairs, the median was 0.052 (IQR 0.134). In the murine HCC tumor, 146 pairs were positively correlated with *r* > 0.30, 8 were negatively correlated with *r* < – 0.30, and the remaining 476 were not substantially correlated with r between −0.30 and 0.30 (Figure 6b). For pairs with *r* > 0.30, the distribution had a median r of 0.427 (IQR 0.141); for pairs with *r* < – 0.30, the median was −0.314 (IQR 0.024); and for the remaining pairs, the median was 0.056 (IQR 0.251). The strongest positive and negative correlations are summarized in Table 2.

**Table 2:** | Top Positive and Negative Correlations between Enzyme Pairs across Tissue Sections

tissue	category	enzyme/activity pair	ion pair	Pearson r
rabbit VX2/iMWA	top positive	DESC1 peptidase–MMP3	cha160_16–cha160_21	0,79
		MMP-2–Neurolysin	cha153_15–cha152_13	0,77
		Cathepsin D–CDC2	cha153_16–cha164.P1	0,68
		Cathepsin D–SIK1	cha153_16–cha193.P1_1	0,64
		Caspase-3–Caspase-7	cha192_15–cha192_20	0,64
	top negative	CDC2–Furin	cha164.P1–cha194_18	–0,56
		Cathepsin D–Furin	cha153_16–cha194_18	–0,56
		Furin–PKC	cha194_18–cha163.P1	–0,55
		Furin–SIK1	cha194_18–cha193.P1_1	–0,55
		Cathepsin S–Furin	cha155_6–cha194_18	–0,53
mouse liver/HCC	top positive	MMP-2–Neurolysin	cha153_15–cha152_13	0,86
		DESC1 peptidase–MMP-3	cha160_16–cha160_21	0,72
		Neurolysin–Plasma Kallikrein	cha152_13–cha152_14	0,65
		Endothelin-converting enzyme 1–Thrombin	cha152_18–cha153_18	0,64
		Endothelin-converting enzyme 1–Neurolysin	cha152_18–cha152_13	0,64
	top negative	KLK5–Parl peptidase	cha152_8–cha167_31	–0,37
		Calpain-1–KLK5	cha151_15–cha152_8	–0,35
		Cathepsin K–Parl peptidase	cha170_22–cha167_31	–0,32
		Cathepsin D–Furin	cha153_16–cha194_18	–0,32
		CDC2–Furin	cha164.P1–cha194_18	–0,31

To further analyze these correlations, undirected enzyme–enzyme networks were constructed from the 36 × 36 Pearson correlation matrices by retaining edges with |*r*| > 0.30. In the iMWA rabbit VX2 liver tumor model, the network comprised 253 edges out of 630 possible pairs (density 0.402), with a greedy modularity Q of 0.168 and 3 modules (sizes 18/13/5; Figure 6c). The highest-degree node was PKC (cha 163.P1_1) (degree 27), followed by Cathepsin D (cha 153_16) (degree 25) and SIK1 (cha 193.P1_1) (degree 24). In the mouse liver, a parallel network analysis revealed a network containing 154 edges (density 0.244), modularity *Q* = 0.267, and 7 modules (sizes 14/14/3/2/1/1/1; Figure 6d). The highest-degree node was SIK1 (cha 193.P1_1) (degree 17), followed by Cathepsin D (cha 153_16) and PKC (cha 163.P1_1) (each 16). A summary of strongly correlated intra- and intermodule pairs is provided in Table 3.

**Table 3:** | Top Correlated Enzyme Pairs Classified by Network Linkage (Intramodule vs Intermodule)

tissue	network linkage	enzyme/activity pair	ion pair	Pearson r
rabbit VX2/iMWA	Intramodule	DESC1 peptidase–MMP-3	cha160_16–cha160_21	0,80
		MMP-2–Neurolysin	cha153_15–cha152_13	0,77
		Cathepsin D–CDC2	cha153_16–cha164.P1	0,68
		Caspase-3–Caspase-7	cha192_15–cha192_20	0,64
	Intermodule	Cathepsin D–Thimet oligopeptidase	cha153_16–cha152_10	0,60
		Cathepsin D–Endothelin-converting enzyme 1	cha153_16–cha152_18	0,59
Mouse liver/HCC	Intramodule	MMP-2–Neurolysin	cha153_15–cha152_13	0,86
		DESC1 peptidase–MMP-3	cha160_16–cha160_21	0,72
		Neurolysin–Plasma Kallikrein	cha152_13–cha152_14	0,65
	Intermodule	KLK5–Plasma Kallikrein	cha152_8–cha152_14	0,62
		Cathepsin D–Thimet oligopeptidase	cha153_16–cha152_10	0,57
		Meprin β–PKA	cha166_21–cha154.P1	0,56

Together, these correlation and network analyses revealed exploratory spatial correlation patterns among enzyme activity maps with differences in network density and modularity between the analyzed tissue models.

### Unsupervised Clustering Distinguishes Tissue Types Based on Enzyme Activity Profiles

To segment spatial activity patterns at 30 min, we used K-means clustering (*k* = 4) on pixelwise ratiometric activity vectors of 36 enzymes. Each pixel is represented by a 36-element vector of ratiometric enzyme activities, and K-means clustering groups pixels with similar multienzyme activity signatures; thus, the clusters arise from similarities across the entire enzymatic profile. In the iMWA rabbit VX2 liver tumor model, clusters 0, 1, 2, and 3 contained 13.5%, 38.6%, 24.9%, and 22.8% of pixels, respectively (Figure 7a). In the mouse model, clusters 0, 1, 2, and 3 included 22.5%, 43.3%, 23.9%, and 10.1% of pixels, respectively (Figure 7b).

**Figure 7:**
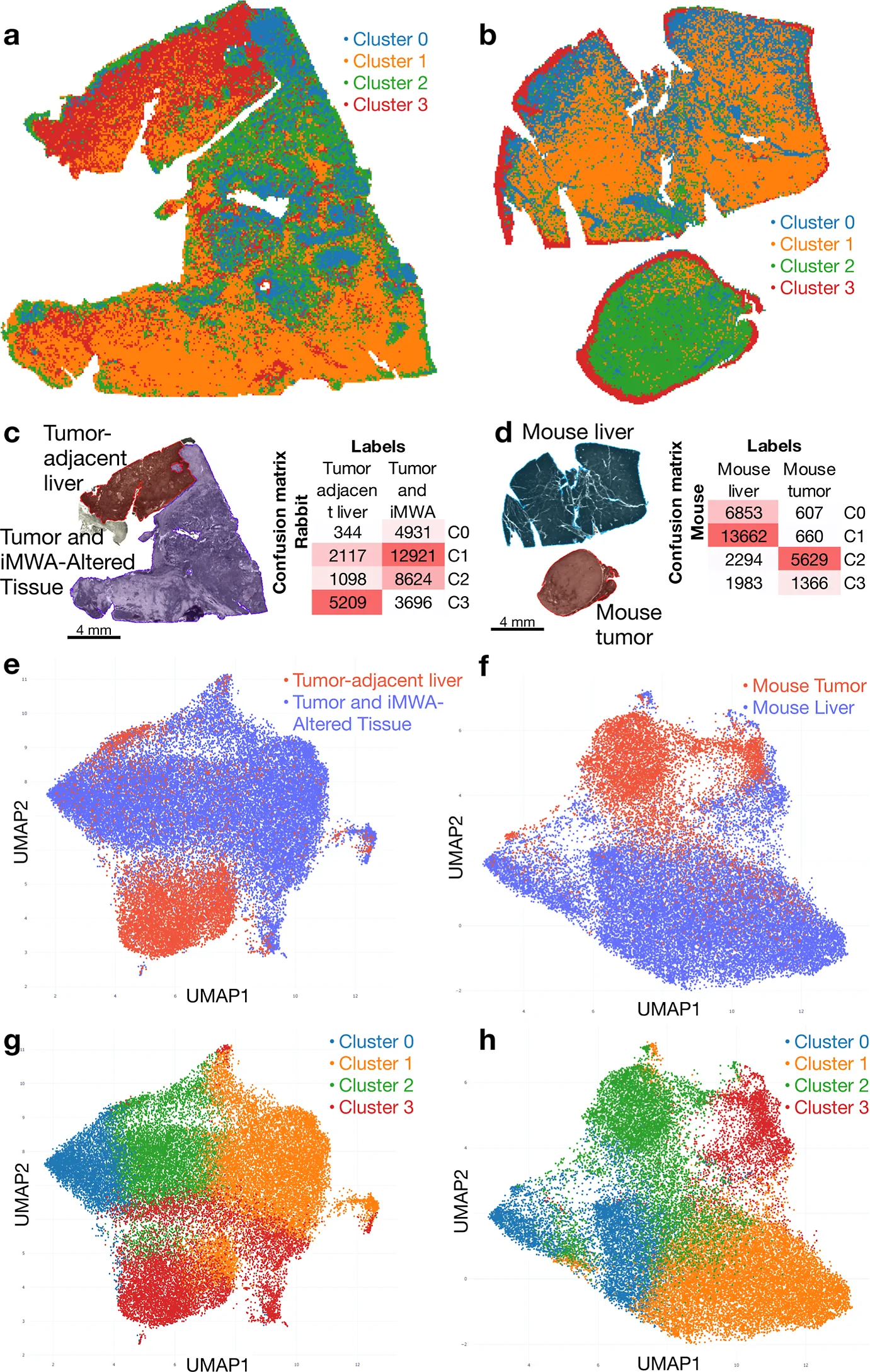
Unsupervised segmentation and embedding of ratiometric enzyme activity at 30 min. (a) K-means cluster map (*k* = 4) for iMWA rabbit VX2 liver tumor-based on 36 product/(product + substrate) ratio features. (b) K-means cluster map (*k* = 4) for the mouse liver model, using the same 36 ratio features as in panel a. (c) Rabbit confusion matrix, with rows as “ tumor-adjacent liver ” and “ Tumor and iMWA-Altered Tissue ” labels and columns represent clusters 0–3. (d) Mouse confusion matrix, where rows represent “Mouse Liver” and “Mouse Tumor” labels and columns as clusters 0–3, formatted identically to panel c. (e) UMAP of rabbit data colored by pathology labels. (h) Mouse UMAP colored by pathology labels. (g) UMAP of rabbit data colored by the corresponding K-means clusters highlighted in panel a. (h) Mouse UMAP colored by the corresponding K-means clusters highlighted in panel b. Interactive HTML versions are provided as supplements.

The clustering results were evaluated against the true labels by using confusion matrices. For the mouse model, mouse liver pixels were primarily grouped in cluster 1, whereas mouse tumor pixels were mostly assigned to cluster 2, although in both cases, some samples were scattered across other clusters. The Hungarian algorithm identified the best label-to-cluster mapping as mouse liver to cluster 1 and mouse tumor to cluster 2 (Figure 7d). Similarly, in the iMWA rabbit VX2 liver tumor model, the tumor-adjacent liver was mainly concentrated in cluster 3, whereas rabbit tumor/iMWA-altered tissue was most abundant in cluster 1, with additional samples spread across other clusters. The optimal mapping obtained using the Hungarian algorithm was tumor-adjacent liver to cluster 3 and tumor/iMWA-altered tissue to cluster 1 (Figure 7c). Together, these results indicate that the clustering effectively captured the main biological separation in both data sets, although some overlap between clusters and labels was observed.

To visualize the global structure of the enzymatic profile, we applied uniform manifold approximation and projection (UMAP) separately to the mouse and rabbit model data. For the rabbit model, the 36-feature ratiometric data at 30 min were embedded in two dimensions. When colored by the true tissue labels (tumor-adjacent liver and tumor/iMWA-altered tissue), the embedding revealed clear groupings matching the biological categories (Figure 7e). When colored by K-means clusters (Figure 7g), a similar separation appeared, with tumor-adjacent liver aligning most closely with cluster 3 and tumor and iMWA-altered tissue distributed across clusters 0, 1, and 2. For the mouse model, a separate UMAP embedding was generated using the same procedure. Visualization by tissue labels (mouse liver and mouse tumor) again showed clear structural separation (Figure 7f), while coloring by the K-means clusters demonstrated strong alignment: mouse liver with cluster 1 and mouse tumor with cluster 2 (Figure 7h). Together, these UMAP plots show that the clustering results are consistent with the underlying biological labels, highlighting the global structure of each data set.

## Discussion

While spatial omics approaches have largely focused on expression profiles, enzymatic activity often differs from abundance and better reflects the functional states of biological programs.^34^ This study established a multiplex, ratiometric MALDI–MSI workflow that measures spatial enzyme activity rather than presence using a 31-peptide library across cancerous rabbit and murine liver tissues. Pixelwise normalization enabled tissue-wide activity maps, correlation networks, and unsupervised structures (K-means and UMAP). This design enables spatial studies of the enzymatic programs that drive tumor progression and microenvironmental remodeling, providing a system-level view of tissue organization that links enzyme activity to tissue architecture and pathological features.

Integration of MEA–MSI with spatial transcriptomics or proteomics through coregistration or adjacent section analysis could enable direct comparison between enzyme abundance and catalytic activity. Furthermore, because the MALDI matrix can be removed after imaging, as done here for H&E staining, it is in principle possible to perform subsequent spatial proteomics measurements on the same tissue section, enabling sequential multimodal analysis. Such approaches may help identify regulatory mechanisms, including post-translational modification or inhibition that decouple the expression from function.

Zinc fixation was selected because previous studies, including our prior work, showed preservation of enzymatic activity in fixed tissue sections.^22,26,35^ In our previous multiplex enzyme activity imaging study, heat inactivation abolished substrate conversion, supporting the interpretation that the detected products arise from enzymatic activity rather than nonenzymatic substrate degradation.^26^ Nevertheless, activity preservation may vary between enzymes, tissue types, fixation batches, and assay conditions, and further optimization of the fixation and incubation parameters may improve reproducibility.

Achieving this functional perspective fundamentally relies on a ratiometric design that additionally counters the effects of ionization bias. Ionization bias constitutes an inherent challenge of MALDI–MSI, with prior efforts of normalization strategies based on external standards (e.g., isotope-labeled, using the chemical noise background or cross-normalization).^36–38^ The ratiometric normalization approach, product/(product + substrate), exploits the concept of mapping the applied enzyme library substrates and their corresponding products. For example, the normalization of the PKA product maps resulted in activity maps that corresponded to the H&E-stained tissue section pattern, in contrast to the raw PKA product ion maps. Limiting the activity maps to one representative product per enzyme reduced the redundancy of multiproduct and multisubstrate reactions, providing inputs for downstream analyses.

In this study, MEA–MSI generated comparable spatial enzyme activity patterns across replicate preparations and independent animals. Ratiometric activity maps were broadly similar between two replicate sections prepared under the same conditions and between two independent animals processed under the matched preparation conditions. In contrast, comparison of the section with replicate preparations generated using a different condition showed both conserved and divergent activity patterns depending on the enzyme. This indicates that the spatial organization of enzyme activity is largely reproducible, whereas the detection of individual substrate and product ions can be affected by preparation-related variables, e.g., matrix, sprayer, or tissue block age. These observations indicate that MEA–MSI in its present form is suited to exploratory multiplexed spatial mapping of enzyme activity, whereas comparison across independently prepared sections requires a standardized sample preparation and further validation.

Activity maps resolved recurrent activity patterns of in vivo-like tissue architecture. In the iMWA rabbit VX2 liver tumor model, activities were segregated across tumor-adjacent liver, a viable tumor with necrotic tissue, and mixed iMWA-altered tissue into different distribution patterns. The tumor/necrotic zone concentrates proteases and kinases classically linked to invasion, apoptosis, and cell cycle regulation, such as MMP-2/-3,^39^ cathepsin D/S,^40–42^ caspase-3,^43^ CDC2,^44^ and PKC,^45^ each of which has been reported to be upregulated or active in tumors and/or aggressive margins. Meprin family metalloproteases and endothelin-converting enzyme-1 in this compartment are consistent with ECM remodeling and pro-invasive endothelin signaling that can be enriched in tumor.^46,47^ Thimet oligopeptidase signal in tumoral regions aligns with reports that the activity of this peptidase is altered in HCC and can participate in tumor-microenvironment peptide processing.^48^ In contrast, enzymes enriched in tumor-adjacent liver or at the tumor interface (e.g., cathepsin L, cathepsin K, and ADAMTS5 peptidase) likely reflect hepatocyte biology. Our observation of tumor-adjacent liver cathepsin L activity is consistent with reports of its staining in hepatocytes;^49^ however, it contrasts with the observations of HCC in the literature, where tumor-cell cathepsin L upregulation is associated with advanced stage, poor differentiation, and recurrence. Additionally, functional studies have shown that overexpression triggers proliferation and tumorigenicity.^50^ These divergent patterns underscore the context dependence of cathepsin L biology, potentially arising from species- or model-specific factors besides microenvironmental or treatment influences. Immune-linked proteases, such as granzyme M and KLK5, were enriched in tumor-adjacent liver and mixed iMWA-altered tissue regions but showed reduced activity within the tumor core, consistent with their immune-related localization at the invasive margins in liver cancer.^51,52^ In mouse liver activity maps, executioner caspases (caspase-3/7) were more active in HCC, consistent with reports that apoptotic executioner signaling is involved in hepatocarcinogenesis and is enriched in tumor tissue or under pro-apoptotic pressure in HCC models.^43^ Matriptase-2 (TMPRSS6), a hepatocyte-derived iron-regulatory protease, is dysregulated in liver cancer. This supports its classification as an HCC-associated signal here, despite some data sets reporting lower TMPRSS6 expression in HCC cell lines than in normal liver.^53^ In contrast, cathepsin K and cathepsin S were predominant in the normal liver in our maps, which diverges from studies linking the upregulation of CTSK and CTSS to pro-tumor/pro-fibrotic programs in liver disease.^54,55^ The biological specificity of the ratiometric MEA–MSI signal was further supported by the ATP-dependence of kinases in a control experiment. When the cosubstrate ATP was added, the enzymatic conversion increased over the whole tissue sections, but especially in regions with high kinase activity. This pattern is consistent with the known ATP requirement of protein kinases, such as PKA and CDC2, for phosphoryl transfer reactions,^56,57^ supporting the biochemical specificity of the MEA–MSI readout. The correlation analysis captured both coordinated and opposing pixel-level activity relationships. In both models, sets of enzymes showed positive associations consistent with the co-occurrence of activity; for example, caspase-3 and caspase-7 exhibited strong correlations reflecting their joint role as executioner caspases that are frequently coactivated during apoptosis, aligning with prior work showing a tight functional coupling of these proteases.^58^ The positive correlation between ECE-1 and thrombin is also mechanistically plausible, as thrombin signaling can upregulate ECE-1 expression and activity, potentially amplifying endothelin signaling in tumor microenvironments.^59^ Network representations derived from |*r*| > 0.30 highlighted species-specific structures: the rabbit network was denser, with fewer and larger modules, whereas the mouse network was sparser, with more and smaller modules and higher modularity. These differences suggest that activity coupling can vary across different models and tissue contexts, even when measured using the same multiplex panel.

Unsupervised analyses provided an orthogonal view of the tissue organization. K-means segmentation of the 30 min, 36-enzyme ratiometric vectors yielded four spatial clusters per section (mouse: 22.5%, 43.3%, 23.9%, and 10.1% of pixels; rabbit: 13.5%, 38.6%, 24.9%, and 22.8%), which corresponded closely to the manually annotated regions after optimal one-to-one mapping by the Hungarian algorithm, mouse liver to cluster 1 and mouse tumor to cluster 3; rabbit tumor-adjacent liver to cluster 3 and rabbit tumor and iMWA-altered tissue to clusters 0, 1, and 2 (Figure 7a–d). The presence of four clusters along with scattered pixels outside the primary clusters for each label in the UMAP projection revealed within-compartment heterogeneity and boundary zones, that is, subregions in tumor and adjacent tissues, as well as gradients across the tumor-adjacent tissue interface that were not captured by binary annotation. These concordances suggest that the dominant gradients in ratiometric enzymatic activity align with histological compartments, while the additional structure revealed by unsupervised methods highlights biologically meaningful heterogeneity that may reflect variable immune/stromal compositions or dynamic remodeling during tumor evolution and treatment. This could mirror the pattern reported in unsupervised spatial transcriptomic studies,^60,61^ but here arises from direct enzymatic activity, underscoring the value of functional mapping for unbiased tissue characterization. In relation to our previous multiplex enzyme activity imaging approach, the present study extends the workflow beyond a mainly protease focused study in healthy kidney tissue to multiple enzyme classes and tumor containing liver models and incorporation of the ratiometric normalization.^26^

Compared to the activity-based imaging approaches outlined in the introduction, MEA–MSI occupies a distinct position in the trade-off between multiplexing and spatial resolution. In situ zymography, fluorogenic and quenched probes, and microscopy-based activity-based protein profiling achieve high sensitivity and, in microscopy formats, subcellular resolution but typically resolve one or a few activities per section due to spectral constraints. MEA–MSI instead provides a readout of multiple enzyme activities across different enzyme classes within a single section at the cost of coarser spatial sampling. Like other enzyme activity-based assays, imaging or nonimaging, MEA–MSI measures the tissue enzyme activities, which can be sensitive to assay conditions, including tissue handling, fixation, incubation time, and detection parameters. Similar dependencies have been reported for in situ zymography and activity-based probe approaches.^13,15,22^ This general condition dependence emphasizes the importance of standardized protocols. Compared with abundance-based spatial methods such as IHC or conventional MSI, it reports catalytic conversion rather than molecular presence.

While these analyses highlight the potential of MEA–MSI, several methodological constraints common to spatial MSI and activity-based mapping must be noted. First, biological replications were comprised of two independent animals per tissue model, together with additional replicate preparations from the original animal, consistent with a proof-of-concept design; accordingly, future experiments should include multiple replicate sections from each animal prepared under matched conditions. The conclusions should be interpreted as the feasibility of mapping rather than definitive quantification. Second, substrate specificity and potential off-target cleavage can complicate the interpretation. These issues are inherent challenges for activity-based assays and mean that MEA–MSI should be interpreted as a broad functional screening and mapping approach. Individual enzyme activities of interest should be confirmed by orthogonal assays. Also, products derived from the same parent substrate are not fully independent because they may share the same substrate pool and differ in formation efficiency and abundance of the target enzyme. Therefore, multiproduct substrates should be interpreted as product-specific apparent activity readouts rather than total parent substrate turnover. Third, detection and analysis thresholds (e.g., OMP feature selection ±0.30 Da; correlation |*r*| > 0.30) inevitably shape the network density and module structure, underscoring the value of sensitivity analyses at alternative cutoffs. Cross-species comparisons remain descriptive, reflecting both architectural and biological differences between models, and should be validated in future studies. More broadly, MEA–MSI signals should be interpreted as apparent enzymatic activity under the defined assay conditions. Product formation reflects substrate conversion and therefore depends on catalytically competent enzyme, but it can also be influenced by enzyme abundance, activation state, endogenous inhibitors, cofactor availability, competing reactions, and tissue preparation. The current study therefore establishes MEA–MSI as a method development and post processing framework for multiplex spatial enzyme activity mapping. This framework enables systematic evaluation of substrate performance, enzymatic conversion, and multiplexing, which will guide optimization of future substrate designs and assay conditions.

These limitations also highlight clear opportunities for extension and refinement. Future developments could improve the biological resolution, analytical depth, and clinical utility of multiplex spatial enzymology in several dimensions. Replicated cohorts and time-course (with more time points) MEA–MSI would refine confidence intervals and quantify dynamic conversion rates, enabling the analysis of apoptotic cascades, signaling kinase turnover, or pathway flux in tumor versus normal compartments. Systematic head-to-head benchmarking against other techniques with respect to reproducibility, throughput, or cost; constitutes an important objective for future work. Expanded peptide libraries with site- and isoform-specific substrates, coupled with inhibitor or competition controls, could disentangle closely related activities. Future substrate designs with more unified chemical properties between substrates and products may further reduce molecule specific ionization efficiency differences and improve quantitative comparability across enzyme readouts. More unified chemical properties and more restrictive modification or cleavage sites may reduce byproduct formation, off-target conversion, substrate competition, and ionization efficiency differences between substrate and product pairs. Increasing spatial resolution (≤20 μm) and coregistration with cell-type maps or spatial transcriptomics/proteomics would enable the attribution of activity programs to specific compartments and cell lineages. Mapping of biopsied tissues, such as human HCC resections, could test the translational utility of multiplex spatial enzymology and benchmark it against expression-based maps.

In summary, MEA–MSI generates spatially resolved activity maps that complement expression-based spatial omics by capturing functional enzymatic pathways. This approach reveals coordinated activity networks and tissue-scale organization across tumor models, providing insights that are inaccessible from abundance alone. The resulting activity maps and analysis outputs constitute reusable resources for the community and a foundation for expanded quantitative studies. In its present form, MEA–MSI offers an exploratory framework for investigating functional tissue states and generating hypotheses about mechanistic studies. Along with future work on optimized substrate designs, workflow standardization, biological replication, and orthogonal confirmation, the method can support quantitative translational and clinical applications.

## Methods

### Chemicals

Fmoc-amino acid TentaGel resins were purchased from Rapp Polymere GmbH (Germany). Fmoc-protected amino acids, *N*,*N*′-diisopropylcarbodiimide (DIC), and Oxyma Pure were purchased from IRIS Biotech GmbH (Germany). All other chemical substances used were of high purity grade (95%–99% pure) and were purchased from Sigma-Aldrich/Merck (Germany) or ROTH (Germany), unless otherwise stated.

### Tissue Sources, Preparation, and Ethics

All procedures involving the use of animals were approved by Charité–Universitätsmedizin Berlin and the State Office for Health and Social Affairs (LAGeSo), Berlin, Germany (protocol numbers T–CH–0015/24, G0048/19, and G150/20). Liver tissues from different animals were processed to prepare zinc-fixed, paraffin-embedded sections for MEA–MSI analysis. Normal liver tissue was obtained from a healthy adult female BALB/c mouse (Janvier Laboratories, France), HCC liver tissue^62^ was obtained from an adult female BALB/c mouse (Charles River, Germany) bearing an orthotopic HCC tumor (TIB-75 cells, ATCC, USA), and an iMWA rabbit VX2 liver tumor (implementation described in detail by Savic et al.^63^) from an adult male New Zealand rabbit (Charles River Laboratories, Germany). Incomplete microwave ablation (iMWA) was performed by placing a 17F microwave needle centrally within the tumor and applying 65 W for 40–60 s to ablate at least 50% of the tumor volume. The abdomen was closed in two layers using absorbable sutures and left for 2 weeks before tissue collection. After euthanasia, the liver tissues were rinsed with sterile 0.9% NaCl solution, cut into pieces with thickness of 3–4 mm and fixed in zinc fixative (BD Pharmingen, Germany) at room temperature for 48 h, washed for 1 h in Millipore water, processed through ethanol (70% for 2 h; 80% for 2 h; 96% for 2 × 2 h; 100% for 2 × 2 h), xylene (100% for 2 × 2 h), and paraffin (2 × 2 h under vacuum, 59 °C), sectioned at 4 μm, and mounted on ITO-coated glass slides (Bruker Daltonik, Germany). Sections were air-dried at room temperature, deparaffinized 100% (3 × 5 min), then treated with xylene/ethanol 1:1 (5 min), rehydrated through ethanol (100% 2 × 5 min; 95%, 70%, and 50%, each 5 min), and dried in a desiccator for 20 min. For each tissue model, the study included two independent animals together with additional replicate preparations from the original animal, to assess cross-animal and technical reproducibility. Deparaffinized sections were then used for MEA library application and MALDI–MSI acquisition.

### Peptide Substrate Syntheses and Quality Control

The multiplex enzyme activity (MEA) library comprised 31 peptide substrates selected from natural protein sequences or prior literature that reported specific cleavage or modification events (full sequences and sources are listed in Table 1 and Supporting Information, Table S1).

The multiplex enzyme activity (MEA) library comprised 31 peptide substrates selected from natural protein sequences and prior literature reporting specific cleavage or modification events. Reported sequences and target sites were cross-checked against enzyme’s substrate databases, including MEROPS for protease cleavage events and PhosphoSitePlus for phosphorylation. Substrates were selected based on reported enzyme recognition motifs, known cleavage or modification sites and suitability for multiplexing without *m*/*z* overlap (full sequence and source information are listed in Table 1 and Supplementary Table S1).

Substrates were synthesized by Fmoc solid-phase peptide synthesis (SPPS) on TCP or TentaGel resin using a Liberty Blue automated synthesizer (CEM Corporation, USA) with DMF as solvent and standard activators (HOBt, DIC, Oxyma Pure). Crude peptides were purified by RP-HPLC (C18(2), 100A°, 250 × 21.2 mm) using an acetonitrile–water gradient, and their identities were confirmed by MALDI–MS in positive-ion mode on a Bruker microflex using CHCA matrix (α-cyano-4-hydroxycinnamic acid) in a dried-droplet spotting protocol. The acceptance criteria required an RP-HPLC purity of ≥95% and a correct monoisotopic mass. Qualified lots were aliquoted at 50 mM in Millipore water (10 μL per aliquot), stored at – 80 °C, and thawed immediately prior to use to minimize freeze–thaw cycles. For pooling, working stocks were diluted to 2 mM in Millipore water per peptide and transferred to the MEA mix for on-tissue incubation.

### MEA Library Preparation and On-Tissue Incubation

The MEA peptide library was prepared to deliver equimolar peptide substrates onto deparaffinized, zinc-fixed liver sections for on-tissue incubation for 30 and 60 min. Working stocks (2 mM per peptide) were pooled to a final concentration of 10 μM per peptide in 20 mM ammonium bicarbonate: acetonitrile (9:1, v/v) supplemented with ATP = 200 μM, acetyl-CoA = 100 μM, UDP-*N*-acetylgalactosamine = 200 μM, and phosphatase inhibitor cocktail 1 (Sigma-Aldrich, at 5% (v/v)). The mixtures were kept on ice and applied using an HTX TM-Sprayer with a nozzle temperature of 30 °C. The MEA was sprayed at a flow rate of 0.015, track speed of 750 mm·min^–1^, 8 passes, line spacing of 2 mm, and spray height of 40 mm. Immediately after deposition, the slides were incubated in a humid chamber at 37 °C for 30 or 60 min and then dried in a desiccator for 10 min. Quality control included an unsprayed negative control region on the same slide shielded with a coverslip. For the ATP-dependence control experiment, adjacent mouse HCC liver sections were incubated with a subset of the substrate library for 30 min with and without ATP and a phosphatase inhibitor cocktail under identical spraying and incubation conditions. The incubated sections were directly subjected to matrix application for MALDI–MSI.

Reproducibility was assessed for each tissue model (mouse normal liver, mouse HCC, and rabbit VX2 iMWA) across replicate preparations and two independent animals. From the original animal, the original section (specimen 1) and two replicate sections (specimens 2 and 3) were prepared. From a second, independent animal of the same tissue type, a further section (specimen 4) was prepared under conditions matched to specimen 1.

### MALDI Matrix Application and MSI Acquisition

Matrix-assisted laser desorption/ionization imaging mass spectrometry (MALDI–MSI) was performed to acquire ion images of the substrates and products from the incubated tissue sections at a spatial sampling of 50 μm. The MALDI matrix was 7% α-cyano-4-hydroxycinnamic acid (CHCA) (Bruker Daltonics) at 70% acetonitrile +1% TFA (v/v) and was applied with an HTX TM-Sprayer set to nozzle temperature = 75 °C, flow rate of 0.12 mL·min^–1^, track speed of 1200 mm·min^–1^, line spacing of 3 mm, and spray height of 40 mm, for 4 passes. Slides with tissue probes were analyzed on a Bruker rapifleX MALDI–TOF in positive-ion mode over *m*/*z* 400–2,500, using a raster step of 50 μm, 500 shots·pixel^–1^, and sampling rate of 1.25 GS·s^–1^; acquisition was controlled with flexControl and flexImaging, and external calibration employed a Bruker Peptide Calibration Standard. Quality control included visual verification of matrix homogeneity and confirmation that the calibrant mass error was ≤0.0001 Da. No TIC or median normalization was applied downstream because ratiometric processing was used. Data were exported as.dat files and imported into SCiLS Lab, where simultaneous preprocessing across the data sets was performed (TopHat baseline removal, width = 20). The resulting data sets were then used for peak finding and ratiometric analysis.

The replicate sections (specimens 2 and 3) were processed using a custom spraying device and a different matrix supplier (Sigma-Aldrich) from the original section. All other parameters, zinc fixation, deparaffinization, substrate concentration, incubation temperature and time, matrix concentration, and MALDI acquisition settings, were identical to the main protocol.

After the MALDI imaging experiments, the matrix was removed with 70% ethanol, and the tissue sections were stained with a hematoxylin–eosin (HE) fast staining kit.

### Data Analysis Pipeline

#### Peak Finding and Ion Detection Criteria; ROC/AUC vs Unsprayed Controls

Feature detection was performed to objectively define the substrate and product ions prior to downstream ratiometric analysis and performance benchmarking. Peaks were identified in SCiLS Lab Software (Bruker Daltonics) using orthogonal matching pursuit (OMP) feature finding on imported spectra, with TopHat baseline removal (width = 20) as a preprocessing step. An ion was classified as detected if its theoretical *m*/*z* matched an OMP-listed feature within an absolute tolerance of ±0.30 Da. The target lists of substrate and product ions are provided in Table 1 and Supplementary Table S2. Discriminative performance was quantified by ROC/AUC comparing sprayed sections (positive class) versus unsprayed controls (covered by glass) using the SCiLS Lab software. The resulting detected/undetected calls, along with the AUC values per ion, were exported from SCiLS Lab (Supplementary Table S2). Per-ion ROC curves, ion intensity maps, and mean/Skyline spectra are provided in Supplementary Figures S1–S4, and the curated ion lists were handed off to the ratiometric computation step.

### Peak Detection, Ratiometric Computation, and Atlas Assembly

Peak finding and ion detection were performed to determine whether each predefined substrate/product *m*/*z* was present and to quantify the discriminative performance versus unsprayed controls. The spectra were processed in SCiLS Lab using orthogonal matching pursuit (OMP) target ions (31 substrates plus products listed in Table 1 and Supplementary Table S2). An ion was classified as detected when its theoretical *m*/*z* matched an OMP feature within ±0.30 Da across the section. Discrimination between sprayed sections (positive class) and unsprayed controls (negative class) was evaluated using ROC/AUC in SCiLS Lab. Regions and spectra were exported as CSV files for downstream analysis. Per-ion detection and AUC values (Supplementary Table S2) and per-ion ROC curves (Supplementary Figures S1–S3) and ion intensity maps are provided in the Supporting Information. Ratiometric activity maps were computed to compensate for pixel-wise readouts against ionization bias and to provide a single representative map per enzyme for analysis. For each substrate-product pair, pixel-wise activity was defined as

2 $$Ratio=\frac{I_{product}}{I_{product}+I_{substrate}}$$

using raw ion intensities without total ion count (TIC) or median normalization, nontissue pixels were masked, and nonfinite ratios were excluded. When two products were observed for protease substrates, one representative product was selected to avoid over-weighting in downstream analyses. In cases where a substrate was recognized by multiple enzymes, distinct products corresponding to the different enzyme activities were retained separately. This procedure yielded 36 representative enzyme maps per tissue for the main analysis, whereas all substrate-product maps at 30 and 60 min were retained for the supplementary figures. The outputs were per-pixel ratio images in the 0–1 range, which were used for atlas creation, correlation/network analysis, and clustering.

Activity maps were assembled to visualize tissue-wide activity patterns in a consistent layout alongside histology. Per-tissue ratio maps were scaled to 0–1 and rendered on the native MSI grid (50 μm) in Python using a fixed Jet color map and identical min–max scaling across enzymes. Region annotations were created on scanned slides (grayscale) using QuPath^64^ and used for figure labels and agreement metrics in the clustering analyses. Statistical comparison of pixel-level ratios between conditions for the ATP-dependence control experiment was performed using a two-sided Mann–Whitney *U* test with GraphPad Prism.

### Correlation Analysis and Hierarchical Clustering

Correlations were computed to quantify enzyme–enzyme correlations across pixels within each tissue and time point using the 36 ratiometric maps. Analyses were performed using Python 3.13 with pandas, NumPy, SciPy, and Plotly libraries. For each tissue, ratio-value data tables were loaded from CSV files, and background pixels were masked. Pearson correlation matrices (36 × 36, 830 unique pairs) were then computed using pandas/NumPy. The correlation matrix was reordered using average-linkage hierarchical clustering, where dissimilarity was considered the correlation distance between feature correlation profiles. Interactive Plotly heat maps were exported as HTML (Supplement), and enzyme correlations were used for network construction and community detection.

### Network Construction and Community Detection

Networks were constructed to summarize the enzyme–enzyme correlation matrices as modules of corelating activities per tissue. Analyses were performed in Python 3.11 using Pandas and NumPy for matrix handling, NetworkX 3.2 for graph operations, and Plotly for visualization. For each tissue-specific 36 × 36 Pearson matrix, an undirected graph was built with one node per enzyme and edges retained where |*r*| ≥ 0.30; edges carried attributes for the signed coefficient (*r*) and absolute weight (|*r*|), and isolated nodes were retained. Several correlation thresholds were evaluated during analysis, and |*r*| ≥ 0.30 was selected as an exploratory cutoff that provided a balance between retaining moderate spatial correlation patterns and avoiding overly dense networks. This threshold was used for descriptive visualization and was not intended to define statistically validated or mechanistic enzyme correlation. Community structure was identified using the greedy modularity algorithm (resolution γ = 1.0), and within-community eigenvector centrality was computed to rank the nodes for plotting. The outputs included interactive chord diagrams (nodes grouped by community, node order by centrality, and intercommunity gaps for clarity) with edge thickness ∝ |*r*| and color by sign (positive = red, negative = blue) exported as HTML.

### K-Means Segmentation and Pathology Agreement Metrics

Unsupervised clustering was performed to segment the recurrent spatial patterns of enzyme activity at 30 min (with 60 min analyses run analogously). Normalized ratio tables were read from the CSV files, and clustering was implemented in Python 3.11 with Scikit-learn 1.4. For each tissue data set (VX2-rabbit, mouse normal, and mouse HCC), pixels were represented by the 36 ratiometric features; missing values were imputed to zero, no additional scaling was applied, K-means was run independently per tissue, and cluster IDs were written back to the MSI grid to produce spatial maps. The model parameters were *k* = 4, n_init = “auto”, and random state = 42. When tissue-level labels were available, agreement was assessed using confusion matrices (rows = labels, columns = cluster IDs), and an optimal one-to-one label-cluster mapping was obtained using the Hungarian assignment.

### UMAP Embedding

UMAP was applied to visualize the global structure of the pixelwise enzyme activity profiles at 30 min. Analyses were implemented in Python 3.11 using UMAP-learn with auxiliary data handling in Pandas and NumPy. Normalized ratio tables were read from the CSV files, missing values were imputed to zero, and UMAP was fit with metric = “euclidean”, n_neighbors = 15, min_dist = 0.10, and random state = 42. The outputs comprised two-dimensional coordinates rendered as interactive HTML colored by tissue and by K-means cluster assignments.

## Supplementary Material











## Notes

Ion maps, skyline, and mean spectra for 30 and 60 min incubations; ROC plots for 30 and 60 min incubations; ratiometric enzyme activity maps (jet colored and raw) for the mouse and rabbit models at 30 and 60 min; H&E-stained sections; correlation heatmaps, correlation networks, and UMAP analyses for the mouse and rabbit models; peptide sequence details; substrate and product detection; and reproducibility comparison (PDF)
Supplement 1 (ZIP)
Supplement 2 (ZIP)
Supplement 3 (ZIP)
Supplement 4 (ZIP)

## Notes

Data and code availability: raw MALDI–MSI data sets and associated metadata (thumbnails, acquisition logs, and calibration files) were deposited in Zenodo under doi.org/10.5281/zenodo.20135370. The analysis notebooks and scripts are hosted on github.com/AG-Molecular-Imaging/MEA–MALDI–MSI. Access conditions and any dates were stated in the repository READMEs, and additional materials (e.g., SCiLS Lab project files) will be provided on request.

## Notes

Availability of data and materials: the data sets used and/or analyzed during the current study are provided as supplements, and the Python code for data analysis is provided at GitHub: github.com/AG-Molecular-Imaging/MEA–MALDI–MSI.
