# Supplementary material for: Multiplexed Enzyme Activity Microscopy by Semi-quantitative, Ratiometric Enzyme Activity MALDI Mass Spectrometry Imaging
Source: ACS Meas Sci Au. 2026 Aug 12;6(5):1507–24. doi: 10.1021/acsmeasuresciau.6c00070 (PMC13693199; doi:10.1021/acsmeasuresciau.6c00070)
Supplement: Supplementary file 1 [file tg6c00070_si_001.pdf]

Multiplexed Enzyme Activity Microscopy by Semiquantitative, Ratiometric Enzyme Activity MALDI Mass Spectrometry Imaging (MEA-MSI)

Hamidreza Hojjat [1],[5], Petra Henklein [2], Akvile Haeckel [1], Grit Nebrich [3], Sylwia Handzik [3], Pedro Augusto Dantas de Moraes [1], Jing Guo [1], Justus Ramtke [1], Yubei He [1], Lynn Jeanette Savic [1],[4], Oliver Seitz [5], Oliver Klein [3]*, Eyk Schellenberger [1]*

1. Institute of Radiology, Charité – Universitätsmedizin Berlin, corporate member of Freie Universität Berlin and Humboldt-Universität zu Berlin, Charitéplatz 1, 10117 Berlin, Germany
2. Institute of Biochemistry, Charité – Universitätsmedizin Berlin, corporate member of Freie Universität Berlin and Humboldt-Universität zu Berlin, Charitéplatz 1, 10117 Berlin, Germany
3. Imaging Mass Spectrometry Unit; Berlin Institute of Health at Charité-Universitätsmedizin Berlin, Augustenburger Platz 1, 13353 Berlin, Germany
4. Berlin Institute of Health at Charité-Universitätsmedizin Berlin, Anna-Louisa-Karsch-Str. 2, 10178 Berlin, Germany
5. Institute of Chemistry, Humboldt-Universität zu Berlin, Brook-Taylor-Str. 2, 12489 Berlin, Germany

* Corresponding authors

Supplementary Information Table of Contents

Supplementary Figures

1. **Supplementary Figure S1.** *Ion maps, Skyline, and mean spectra for 30-minute incubation.*
   - Visualization of peptide ion distribution, skyline and average spectra following 30 min incubation.
2. **Supplementary Figure S2.** *Ion maps, Skyline, and mean spectra for 60-minute incubation.*
   - Visualization of peptide ion distribution, skyline and average spectra following 60 min incubation.
3. **Supplementary Figure S3.** *ROC plots for 30-minute incubation.*
   - ROC curves evaluating discrimination between test and control groups following 30 min incubation
4. **Supplementary Figure S4.** *ROC plots for 60-minute incubation.*
   - ROC curves evaluating discrimination between test and control groups following 60 min incubation
5. **Supplementary Figure S5.** *Mouse model, 30-minute incubation.*
   - Jet coloured, ratiometric enzyme activity maps after 30 min of incubation in the mouse liver model.
6. **Supplementary Figure S6.** *Rabbit model, 30-minute incubation.*
   - Jet coloured, ratiometric enzyme activity maps after 30 min of incubation in the rabbit model.
7. **Supplementary Figure S7.** *Mouse model, 60-minute incubation.*
   - Jet coloured, ratiometric enzyme activity maps after 60 min of incubation in the mouse liver model.
8. **Supplementary Figure S8.** *Rabbit model, 60-minute incubation.*
   - Jet coloured, ratiometric enzyme activity maps after 60 min of incubation in the rabbit model.
9. **Supplementary Figure S9.** *Raw data — Mouse model, 30-minute incubation.*
   - Unprocessed ratiometric enzyme activity maps after 30 min of incubation in the mouse liver model.
10. **Supplementary Figure S10.** *Raw data — Rabbit model, 30-minute incubation.*
    - Unprocessed ratiometric enzyme activity maps after 30 min of incubation in the rabbit model.
11. **Supplementary Figure S11.** *Raw data — Mouse model, 60-minute incubation.*
    - Unprocessed ratiometric enzyme activity maps after 60 min of incubation in the mouse liver model.
12. **Supplementary Figure S12.** *Raw data — Rabbit model, 60-minute incubation.*

- Unprocessed ratiometric enzyme activity maps after 60 min of incubation in the rabbit model.

13. **Supplementary Figure S13.** *H&E staining for 30 min incubation section.*
14. **Supplementary Figure S14.** *H&E staining for 60 min incubation section.*
15. **Supplementary Figure S15.** *H&E staining for Specimens 2.*
16. **Supplementary Figure S16.** *H&E staining for Specimens 3.*
17. **Supplementary Figure S17.1 and S17.2.** *H&E staining for Specimens 4.*
18. **Supplementary Figure S18.** *Mouse model(S2), 30-minute incubation.*
    - Jet coloured, ratiometric enzyme activity maps after 30 min of incubation in the mouse liver model
19. **Supplementary Figure S19.** *Mouse model(S3), 30-minute incubation.*
    - Jet coloured, ratiometric enzyme activity maps after 30 min of incubation in the mouse liver model
20. **Supplementary Figure S20.** *Mouse model(S4), 30-minute incubation.*
    - Jet coloured, ratiometric enzyme activity maps after 30 min of incubation in the mouse liver model
21. **Supplementary Figure S21.** *Rabbit model(S2), 30-minute incubation.*
    - Jet coloured, ratiometric enzyme activity maps after 30 min of incubation in the rabbit model.
22. **Supplementary Figure S22.** *Rabbit model(S3), 30-minute incubation.*
    - Jet coloured, ratiometric enzyme activity maps after 30 min of incubation in the rabbit model.
23. **Supplementary Figure S23.** *Rabbit model(S4), 30-minute incubation.*
    - Jet coloured, ratiometric enzyme activity maps after 30 min of incubation in the rabbit model.

Supplementary Correlation Heatmaps

1. **Supplementary Correlation Heatmap S1.** *Mouse model, 30-minute incubation (short list).*
    - Correlation matrix of selected enzymes in mouse tissue after 30 min incubation.
2. **Supplementary Correlation Heatmap S1.** *Mouse model, 30-minute incubation (full).*
    - Full correlation matrix of complete list of enzymes for mouse 30 min dataset.
3. **Supplementary Correlation Heatmap S2.** *Rabbit model, 30-minute incubation (short list).*
    - Correlation matrix of selected enzymes in rabbit tissue after 30 min incubation.
4. **Supplementary Correlation Heatmap S2.** *Rabbit model, 30-minute incubation (full).*
    - Full correlation matrix of complete list of enzymes for rabbit 30 min dataset.
5. **Supplementary Correlation Heatmap S3.** *Mouse model, 60-minute incubation.*
    - Correlation matrix of selected enzymes in mouse tissue after 60 min incubation.
6. **Supplementary Correlation Heatmap S4.** *Rabbit model, 60-minute incubation.*
    - Full correlation matrix of complete list of enzymes for rabbit 60 min dataset.
7. **Supplementary Correlation Heatmap S5.** *Mouse model, 60-minute incubation (short list).*

    - Correlation matrix of selected enzymes in mouse tissue after 60 min incubation.
8. **Supplementary Correlation Heatmap S6.** *Rabbit model, 60-minute incubation (short list).*
    - Full correlation matrix of complete list of enzymes for rabbit 60 min dataset.

Supplementary Correlation Networks

1. **Supplementary Correlation Network S7.** *Mouse model, 30-minute incubation (short list).*
    - Chord network visualization of correlated analytes in mouse 30 min dataset.
2. **Supplementary Correlation Network S8.** *Rabbit model, 30-minute incubation (short list).*
    - Chord network visualization of correlated analytes in rabbit 30 min dataset.

Supplementary UMAP Analyses

1. **Supplementary UMAP S9.** *Mouse model, 30-minute incubation (K-means labels).*
    - UMAP projection of mouse data labeled by K-means clustering.
2. **Supplementary UMAP S10.** *Mouse model, 30-minute incubation (tissue labels).*
    - UMAP projection of mouse data colored by tissue labels.
3. **Supplementary UMAP S11.** *Rabbit model, 30-minute incubation (K-means labels).*
    - UMAP projection of rabbit data labeled by K-means clustering.
4. **Supplementary UMAP S12.** *Rabbit model, 30-minute incubation (tissue labels).*
    - UMAP projection of rabbit data colored by tissue labels.

Supplementary Tables

1. **Supplementary Table S1.** *Peptide sequence details.*
2. **Supplementary Table S2.** *Substrate and product detection and AUC values.*
3. **Supplementary Table S3.** *Substrate and product detection (Detection across the original animal and its replicate preparations, Specimens 1–3).*
4. **Supplementary Table S4.** *Substrate and product detection (Detection across two independent animals, Specimen 1 vs Specimen 4).*
